# Development of a necroptosis-related gene signature and the immune landscape in ovarian cancer

**DOI:** 10.1186/s13048-023-01155-9

**Published:** 2023-04-25

**Authors:** Sipei Nie, Na Ni, Ningxin Chen, Min Gong, Ercui Feng, Jinhui Liu, Qiaoling Liu

**Affiliations:** 1 Department of Gynecology and Obstetrics, Affiliated Jiangning Hospital of Nanjing Medical University, Nanjing, 211100 Jiangsu China; 2 Department of Preventive Health Care, Affiliated Jiangning Hospital of Nanjing Medical University, Nanjing, 211100 China; 3 Department of Gynecology, the First Affiliated Hospital of Nanjing Medical University, Nanjing, 211100 Jiangsu China

**Keywords:** Ovarian cancer, Bioinformatics analysis, Necroptosis-related gene, Prognosis, Tumor microenvironment

## Abstract

**Background:**

Necroptosis is a novel type of programmed cell death distinct from apoptosis. However, the role of necroptosis in ovarian cancer (OC) remains unclear. The present study investigated the prognostic value of necroptosis-related genes (NRGs) and the immune landscape in OC.

**Methods:**

The gene expression profiling and clinical information were downloaded from the TCGA and GTEx databases. Differentially expressed NRGs (DE-NRGs) between OC and normal tissueswere identified. The regression analyses were conducted to screen the prognostic NRGs and construct the predictive risk model. Patients were then divided into high- and low-risk groups, and the GO and KEGG analyses were performed to explore bioinformatics functions between the two groups. Subsequently, the risk level and immune status correlations were assessed through the ESTIMATE and CIBERSORT algorithms. The tumor mutation burden (TMB) and the drug sensitivity were also analyzed based on the two-NRG signature in OC.

**Results:**

Totally 42 DE-NRGs were identified in OC. The regression analyses screened out two NRGs (MAPK10 and STAT4) with prognostic values for overall survival. The ROC curve showed a better predictive ability in five-year OS using the risk score. Immune-related functions were significantly enriched in the high- and low-risk group. Macrophages M1, T cells CD4 memory activated, T cells CD8, and T cells regulatory infiltration immune cells were associated with the low-risk score. The lower tumor microenvironment score was demonstrated in the high-risk group. Patients with lower TMB in the low-risk group showed a better prognosis, and a lower TIDE score suggested a better immune checkpoint inhibitor response in the high-risk group. Besides, cisplatin and paclitaxel were found to be more sensitive in the low-risk group.

**Conclusions:**

MAPK10 and STAT4 can be important prognosis factors in OC, and the two-gene signature performs well in predicting survival outcomes. Our study provided novel ways of OC prognosis estimation and potential treatment strategy.

**Supplementary Information:**

The online version contains supplementary material available at 10.1186/s13048-023-01155-9.

## Introduction

Ovarian cancer (OC) is the most deadly gynecological malignancy, and the number of newly diagnosed cases is increasing worldwide [1]. Regrettably, due to ineffective early screening, most OC patients are diagnosed at an advanced stage [2]. The long-term survival rate remains poor at 30% [3]. The mechanism of OC initiation and progression remains unclear. Clinical predictors, such as cancer antigen 125, have been investigated to evaluate chemotherapeutic efficacy and prognosis but show poor accuracy [4]. Constructing high-quality cancer prediction models incorporating gene panels for individualized diagnosis or prognosis are urgently required.

Necroptosis, also known as programmed necrosis, was discovered as a novel programmed form of necrotic cell death that bears a mechanistic resemblance to apoptosis and a morphological resemblance to necrosis [5]. Necroptosis is mainly initiated by activating various surface-associated death receptors (DR), such as tumor necrosis factor receptor 1 and DR4/5 [6]. RIPK1, RIPK3, MLKL, and necrostatin-1 have been demonstrated to be the main characters in mediating necroptosis [7]. Increasing evidence has suggested that necroptosis plays a significant role in various diseases, such as neurodegenerative diseases, ischemic cardiovascular, and malignancies [8]. Recent studies have revealed the pivotal role of necroptosis in cancer biology regulation, including cancer initiation, cancer metastasis, cancer immunity, and cancer subtypes [9, 10]. Necroptosis has gradually been recognized as a promising therapeutic strategy, and research about cancer-targeting therapy based on necroptosis is currently underway [11]. Necroptosis was found dual-sided in malignancies. On the one hand, it acts as a protective mechanism to prevent cancer progression and promote cancer treatment-induced cell-programmed death. For example, ectopic activation of RIPK3 was suggested to act as a cancer suppressor to inhibit malignant mesothelioma progression by inducing necrotic apoptosis. DNA methylation of RIPK3 impaired necroptosis and led to chemoresistance and poorer prognosis in malignant mesothelioma [12]. Annkathrin Koch et al. demonstrated that blocking MLKL-mediated necroptotic signaling could protect Burkitt’s lymphoma cells from TBZ-treatment-induced cell-programmed death [13]. On the other hand, necroptosis could also accelerate cancer development [14]. Previous studies showed that DR6 is involved in cancer cell-induced endothelial necroptosis, leading to extravasation and metastasis [15]. Lately, mounting evidence has shown that necroptosis is highly involved in various cellular processes of OC, such as chemoresistance and immune response [16]. Dey et al. demonstrated that BMI1 could participate in the PINK1-PARK2-dependent mitochondrial pathway and induce a novel necroptosis-mediated cell death pattern in OC [17]. In addition, inhibition of caspase-8 was found to significantly inhibit NF-κB signaling and lead to necrotic cell death by stabilizing RIPK1 expression in OC [18]. So far, the specific mechanism of necroptosis in the OC tumor microenvironment (TME) remains unclear.

**Fig. 1:**
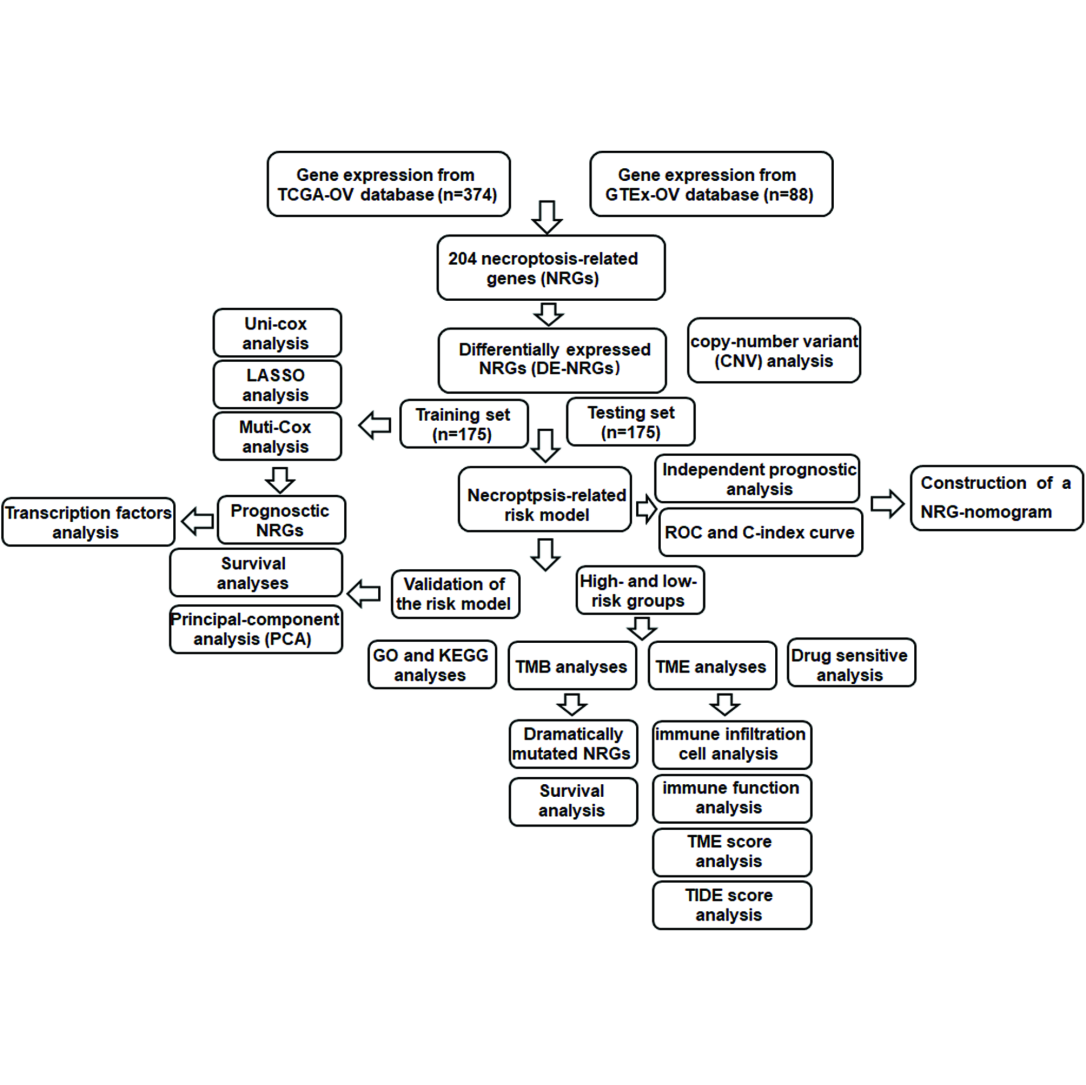
The strategy of the present study

In the present study, we analyzed the differentially expressed genes (DEGs) based on the expression levels of necroptosis-related genes in The Cancer Genome Atlas (TCGA) and Genotype-Tissue Expression (GTEx) databases. We identified the differentially expressed necroptosis-related DEGs (DE-NRGs) in OC. Next, a prognostic model was established by regression analyses of NRGs to evaluate the risk score of OC patients and divided them into high- and low-risk groups. The clinical characteristics and drug sensitivities in the two groups were further investigated. Moreover, we explored the role of necroptosis in TME of OC to provide new ideas for immunotherapy of OC. The strategy of our study is shown in Fig. 1.

## Materials and methods

### Publicly attainable expression datasets

RNA sequencing profiles of OC (n = 379) and normal ovarian epithelial tissue (n = 88) were respectively downloaded from TCGA and GTEx databases in the UCSC Xena platform (https://xenabrowser.net/datapages/) [19–21]. After excluding patients lacking RNA sequencing, 374 samples were kept for further study. Corresponding gene mutation data, copy-number variant (CNV), and related clinical information in TCGA were also downloaded. Among these 374 EOC patients, a total of 48 patients with follow-up time < 90 days (17 patients with follow-up time < 30 days). Patients with short OS values (< 90 days) were excluded to reduce statistical bias. All analyses were performed with R 4.2.1.

### Identification of DE-NRGs

We searched the previous literature and obtained 204 necroptosis genes in Supplementary Table 1. Then 42 DE-NRGs were identified using the “limma” package (Log2 | fold change (FC) | >1 and false discovery rate (FDR) < 0.05). Furthermore, the CNV of DE-NRGs, which significantly enriches the diversity of genetic variation in the genome, was further investigated to exhibit genes with significant amplification or deletion.

### Establishment and validation of prognostic risk assessment model

The 374 individuals were divided into training and testing cohorts in a 1:1 ratio using the R package “caret”. All 42 DE-NRGs were inputted in the training cohort to perform univariate Cox (uni-Cox) regression with a p-value < 0.05. Then, the least absolute shrinkage and selection operator (LASSO) regression analysis was performed. Further, NEGs screened by the LASSO analysis were used for multivariate Cox (multi‐Cox) proportional hazards regression and NRG-signature construction. The risk score was calculated using the following formula: risk score = Σ (expression gene) × coefficient(gene) [22]. The median risk score was used to stratify patients into high‐ and low-risk groups in training and testing cohorts. The R packages “survival” and “survminer” were introduced to evaluate OS using Kaplan Meier (K-M) method. We plotted the K-M, risk, and survival status curves of the training, testing, and entire set using the “pheatmap” R package. To further test the reliability of this model, survival analyses were conducted in subgroups according to generic clinicopathological variables. Patients were divided into subgroups of age < 60/ age ≥ 60 (the median age of patients was 60 years old). Besides, patients were divided into subgroups of grade 1–2/ grade 3 and stage I-II/ stage III-IV according to the tumor grade and FIGO stage.

### Independent prognostic analysis and construction of the nomogram

The correlation between the clinical features and the risk score was validated by the chi-square test. The independent risk factors, including age, tumor grade, tumor stage, and risk score, were assessed by uni‐Cox and multi‐Cox regression analyses. We subsequently applied the receiver operating characteristic (ROC) curves and concordance index(C‐index) to measure the prognostic value of the signature by using the “survival”, “timeROC”, and “rms” packages. We further constructed a nomogram based on the risk score and clinical characteristics. The nomogram performance was evaluated by the calibration curve at 1, 3, and 5 years [23].

### Transcription factors correlation analysis

Transcription factors (TFs) correlation analysis was performed to understand the regulatory mechanism of the prognostic NRGs. A total of 318 TFs were obtained from the cistrome Cancer database (http://cistrome.org/CistromeCancer/CancerTarget/) for subsequent research [24]. we first screened differentially expressed TFs (DE-TFs) in OC (Log2 |FC|>1 and FDR < 0.05). Then we investigate the correlation test between DE-TFs and prognostic NRGs. The correlation coefficient and p-value were calculated by the “cor. test” in R, whose core method was the Pearson test.

### Principal-component analysis

In order to investigate the prominent distinction between the high- and low-risk groups. Principal-Component Analysis (PCA) was conducted based on expression profiles of all genes, DE-NRGs, and the prognostic NRGs contributing to the risk assessment model [25]. The “limma” and “scatterplot3d” R packages were used in PCA.

### Functional enrichment analysis and tumor mutation burden analysis

In order to illuminate the relevant function enrichments between the high-and low-risk group, we identified the DEGs among the two groups (p-value < 0.05 and log2|(FC)|>1). Subsequently, Gene Ontology (GO) analysis and Kyoto Encyclopedia of Genes and Genomes (KEGG) enrichment analysis were performed using the “clusterProfiler” in R, and terms with a p-value < 0.05 and q-value < 0.05 were indicated significantly enriched [26, 27]. Furthermore, we performed the “maftools” package to show tumor mutation burden (TMB) in the high- and low-risk group. Then, we investigate the prognosis of TMB in OC.

### Comprehensive analysis of the TME and immune cell infiltration

Based on the results of functional enrichment analyses, we evaluated the TME score of OC patients in the high- and low-risk groups using the “ESTIMATE” in R [28]. ESTIMATE is a computerized algorithm that calculates the presence of stromal cells (immune score), presence of stromal cells (stromal score), and tumor purity (ESTIMATE score) of each sample for preliminary evaluation. Subsequently, The CIBERSORT algorithm was introduced to estimate the proportions of 22 immune cells in OC samples [29]. Spearman’s correlation was calculated between the immune cell proportions and the risk score. Moreover, Tumour Immune Dysfunction and Exclusion (TIDE) (http://tide.dfci.harvard.edu/) algorithm was used to evaluate immune checkpoint response in the high- and low-risk groups [30]. It was known that a higher TIDE score presented a poorer immune checkpoint inhibitor (ICI) treatment and shorter survival. The p-value < 0.05 was considered statistically significant.

### Drug sensitivity analysis

The chemotherapy response of OC patients was evaluated through the Genomics of Drug Sensitivity in Cancer database (GDSC, https://www.cancerrxgene.org) [31]. The half-maximal inhibitory concentration (IC50), which represented the drug response, was calculated by using the “pRRophetic” R package [32]. The p-value < 0.001 and correlation > 0.4 was considered statistically significant.

## Results

### Identification of DE-NRGs and the CNV of DE-NRGs

**Fig. 2:**
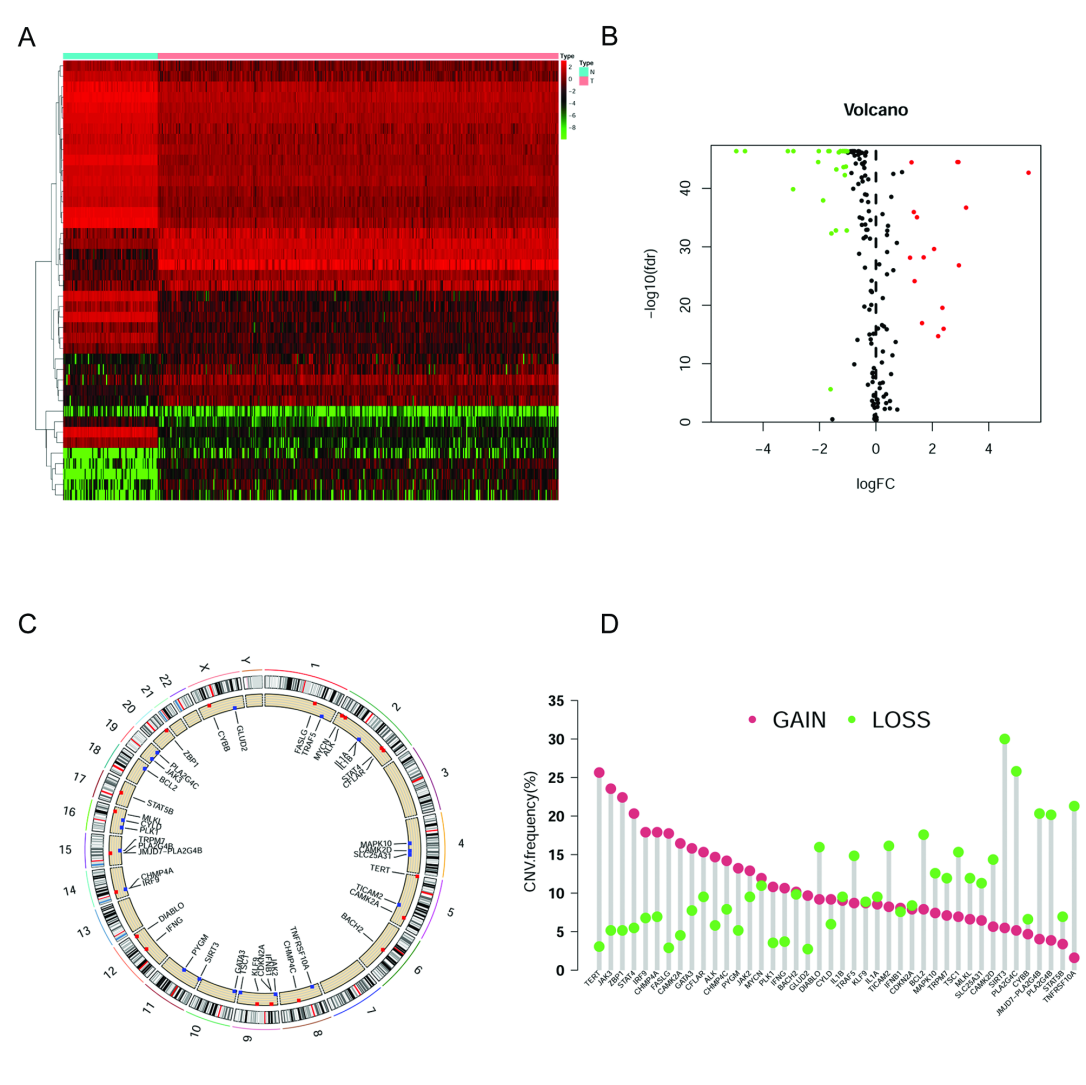
Expressions of the 42 DE-NRGs and the gene CNV analysis. **(A and B)** The heatmap and vioplot of 42 DE-NRGs in 374 OC and 88 normal ovarian tissues (FDR < 0.05 and log2|FC| > 1). **(C)** CNV frequencies of 42 DE-NRGs in OC. **(D)** Circus plots of chromosome distributions of DE-NRGs

Based on DEGs between OC and normal ovarian tissues, 42 DE-NRGs were identified (16 up-regulated genes, 26 down-regulated genes; Fig. 2A and B, Supplementary Table 2). These genes may be used to determine independent prognostic markers in OC. We further explore DE-NRGs exhibiting significant amplification or deletion based on the CNV information because of their potential clinical implications. Circus plots of chromosome distributions of DE-NRGs were shown in Fig. 2C, and we found that most of the DE-NRGs had high frequencies of CNVs in OC (Fig. 2D). We suspected that CNV could be a dominating factor in perturbating the expression of the above DE-NRGs. In the present study, DE-NRGs with a high frequency (> 20%) of amplification were TERT, JAK3, ZBP1, and STAT4, and genes with a high frequency of deletion were SIRT3, PLA2G4C, TNFRSF10A, JMJD7-PLA2G4B, and PLA2G4B.

### Generation and validation of a necroptosis-related risk model in OC

**Fig. 3:**
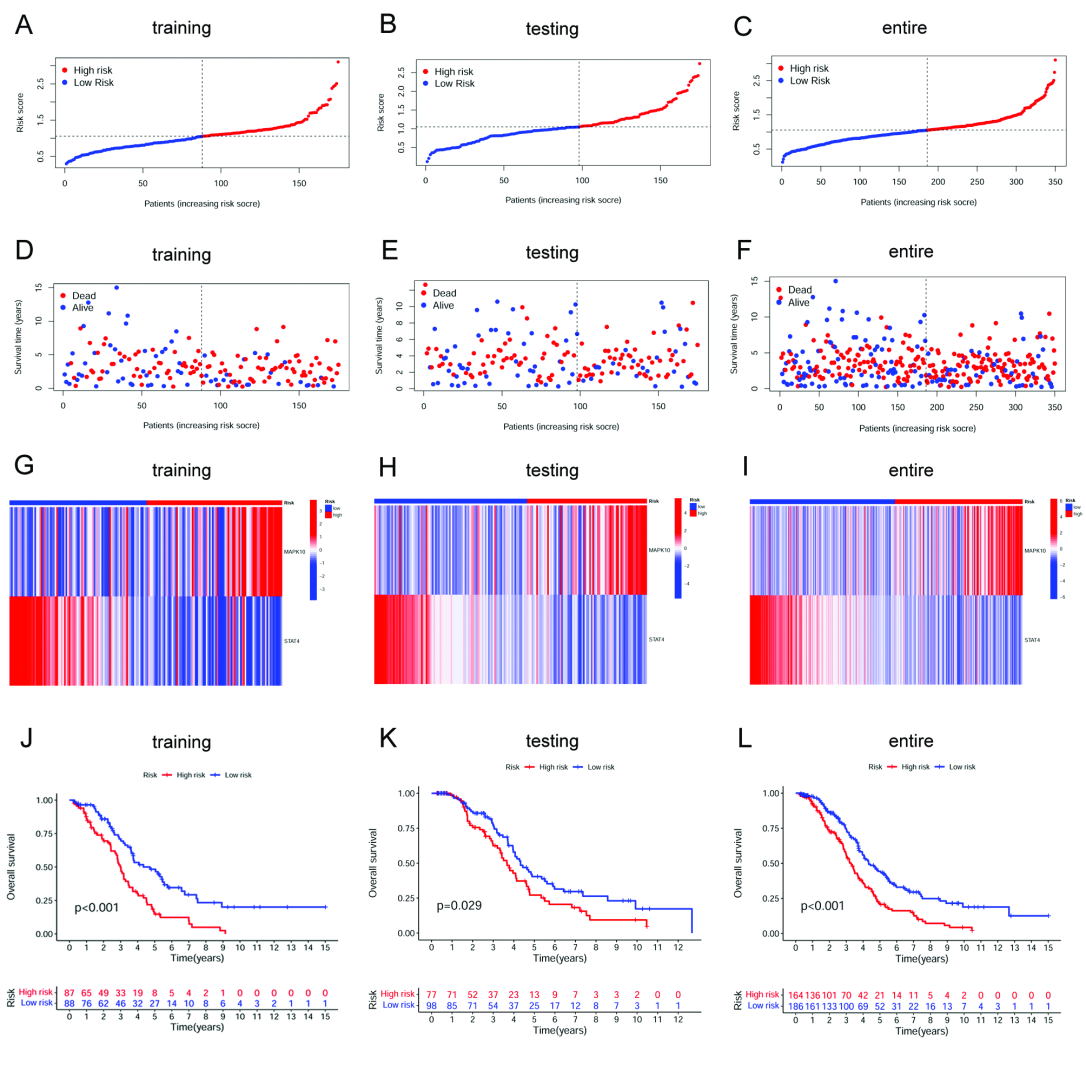
Construction and validation of the NRG signature for OC. **(A-C)** Distribution of OC patients in high- and low-risk groups stratified by the NRG signature in the training, testing, and entire set. **(D–F)** Survival statuses of patients in different groups stratified by the NRG signature in the training, testing, and entire set. **(G–I)** Heatmap of two extracted NRGs expression in the training, testing, and entire set. **(J-L)** Kaplan–Meier survival analysis curves in the training, testing, and entire TCGA-OV set

**Fig. 4:**
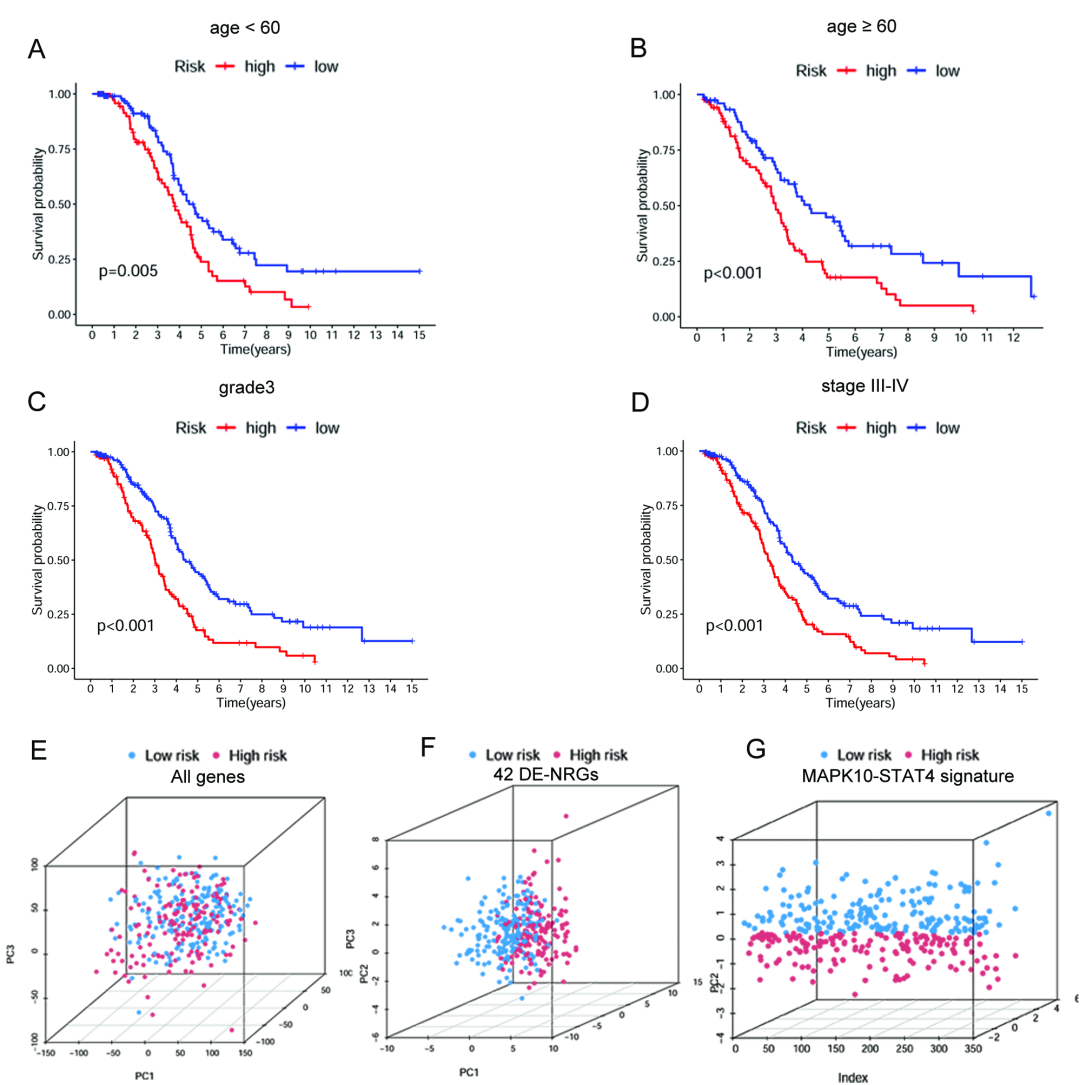
Survival analyses in subgroups **(A)** age < 60, **(B)** age ≧ 60, **(C)** grade 3, and **(D)** stage III-IV. **(E)** PCA of the high- and low-risk groups stratified by the whole-genome **(F)**, NRGs **(G)**, and the NRG signature

Firstly, to develop the necroptosis-related signature to predict the survival outcomes of OC patients, a total of 350 OC patients with a survival time > 90 days were randomly grouped into a training set (175 patients) and a testing set (175 patients) according to a 1:1 ratio. The uni-Cox regression was performed based on the 42 DE-NRGs in samples of the training cohort. As a result, seven NRGs were found to be associated with OC patients’ OS (p-value < 0.05) (Supplementary Fig. 1A). Furthermore, we used the LASSO regression analysis on the obtained prognostic genes to select the best group of prognostic NRGs (Supplementary Fig. 1B and 1C). Eventually, two of them were introduced into the multi-Cox analysis and established the risk model. The results showed that the two NRGs could act as prognostic predictors when coupled with the multi-Cox regression coefficient value. The risk score was calculated as risk score = (0.6683) * MAPK10+ (-1.4049) * STAT4. Each sample’s risk score was calculated according to the formula in the training, testing, and entire set. Next, the median of risk scores was used as the determined cutoff value to group patients into the low- or high-risk group (Fig. 3A-C). The distribution of survival status in each set is displayed in Fig. 3D-F. The expression levels of MAPK10 and STAT4 were also shown in heatmaps in each set (Fig. 3G-I). The survival analysis results showed that patients in the low-risk group were associated with better survival (Fig. 3J-L). These findings revealed that the increased risk score was positively correlated with a poorer prognosis. Moreover, OC patients were grouped by generic clinicopathological variables, including age, tumor grade, and tumor stage. Survival analyses were conducted in subgroups. The results showed that the OS rate was much higher in the low-risk group in patients with age < 60, age ≥ 60, grade 3, and stage III-IV (Fig. 4A-D). The results suggested that this risk model can help predict the prognosis of OC patients with different clinicopathological features. The results of PCA showed separation between the patients in the low- and high-risk groups based on the expression of all genes, the 42 DE-NRGs, and the MAPK10-STAT4 signature (Fig. 4E-G). The outcomes indicated that the two-gene signature possessed the best discriminatory ability to distinguish the low- and high-risk samples.

### Independent prognostic analysis and construction of the nomogram

**Fig. 5:**
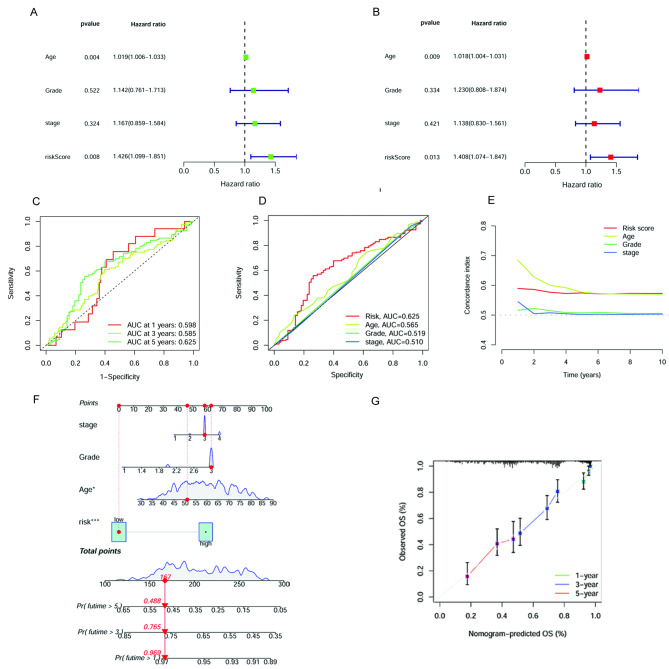
Verification of prognosis gene signature. **(A**) Uni-Cox analysis of clinicopathologic features and the risk score for OS. **(B)** Multi-Cox analysis of clinicopathologic features and the risk score for OS. **(C)** The ROC curves for 1-, 3- and 5-year OS. **(D)** The ROC curves for the 5-year OS of risk score and the other clinical features. **(E)** The C-index curves of risk score and the other clinical features. **(F)** Nomogram for predicting overall survival. **(G)** The calibration curves for 1-, 3- and 5-year OS

The correlation between the clinical characteristics and the risk group was validated by a chi-square test (Supplementary Table 3). Then, the uni-Cox and multi-Cox analyses were conducted, and the results showed that the age and the necroptosis-related risk score could act as independent risk factors for OC patients (Fig. 5A and B). As the AUC of the ROC curve showed, the risk score had higher 5-year OS prediction accuracy than the other clinical factor (AUC = 0.625) (Fig. 5 C and 5D). The 10-year C-index of risk score and age showed higher than that in the grade and stage (Fig. 5E). Combining the risk score and clinical variables, we constructed a nomogram to estimate 1-, 3-, and 5-year OS for OC patients (Fig. 5F). The calibration curves of the nomogram displayed a high consistency between the observed and prognostic values (Fig. 5G).

### Tumor mutation burden and survival analysis in high- and low-risk groups

**Fig. 6:**
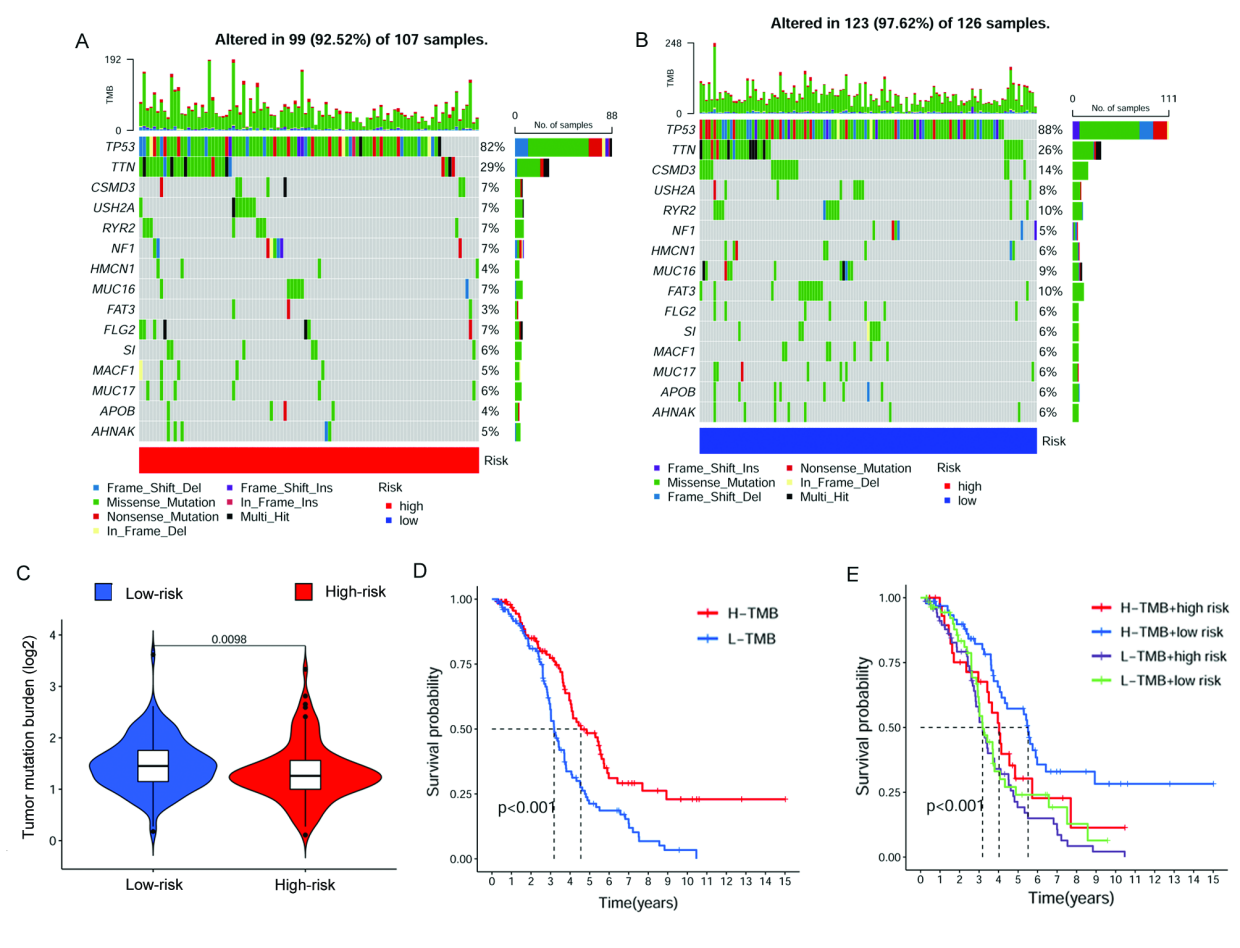
TMB and Chemotherapeutic Sensitivity. **(A-B)** The waterfall plot of somatic mutation features in high- and low-risk groups. **(C)** TMB between the high- and low-risk groups. **(D)** K–M survival analysis combined the risk level and the TMB

The somatic mutation data were obtained from the TGCA database to investigate TMB in the high- and low-risk groups. The ten most dramatically mutated NRGs were TP53, TTN, CSMD3, USH2A, RYR2, NF1, HMCN1, MUC16, FAT3, FLG2, SI, MACF1, MUC17, APOB and AHNAK. Among these genes, TP53 and TTN were the most frequently mutated NRGs in OC (Fig. 6A and B). The result showed that TMB is higher in the low-risk group (Fig. 6C). Besides, survival analyses revealed that patients with high-TMB were related to a better prognosis, and patients with high TMB and low-risk score possessed the best survival outcome than the other groups (Fig. 6D and E).

### The biological pathways analyses in high- and low-risk groups

After exploring the DEGs between high- and low-risk groups, the biological pathways analyses were conducted. The GO analysis showed that the DEGs were strongly enriched in immune-related biological function (Supplementary Fig. 2). At the same time, the results of KEGG analysis also revealed that NRGs were associated with Th1 and Th2 cell differentiation, Antigen processing and presentation, Th17 cell differentiation, Cytokine − cytokine receptor interaction, and Natural killer cell-mediated cytotoxicity pathways.

### Exploration of immune landscape in high- and low-risk groups

**Fig. 7:**
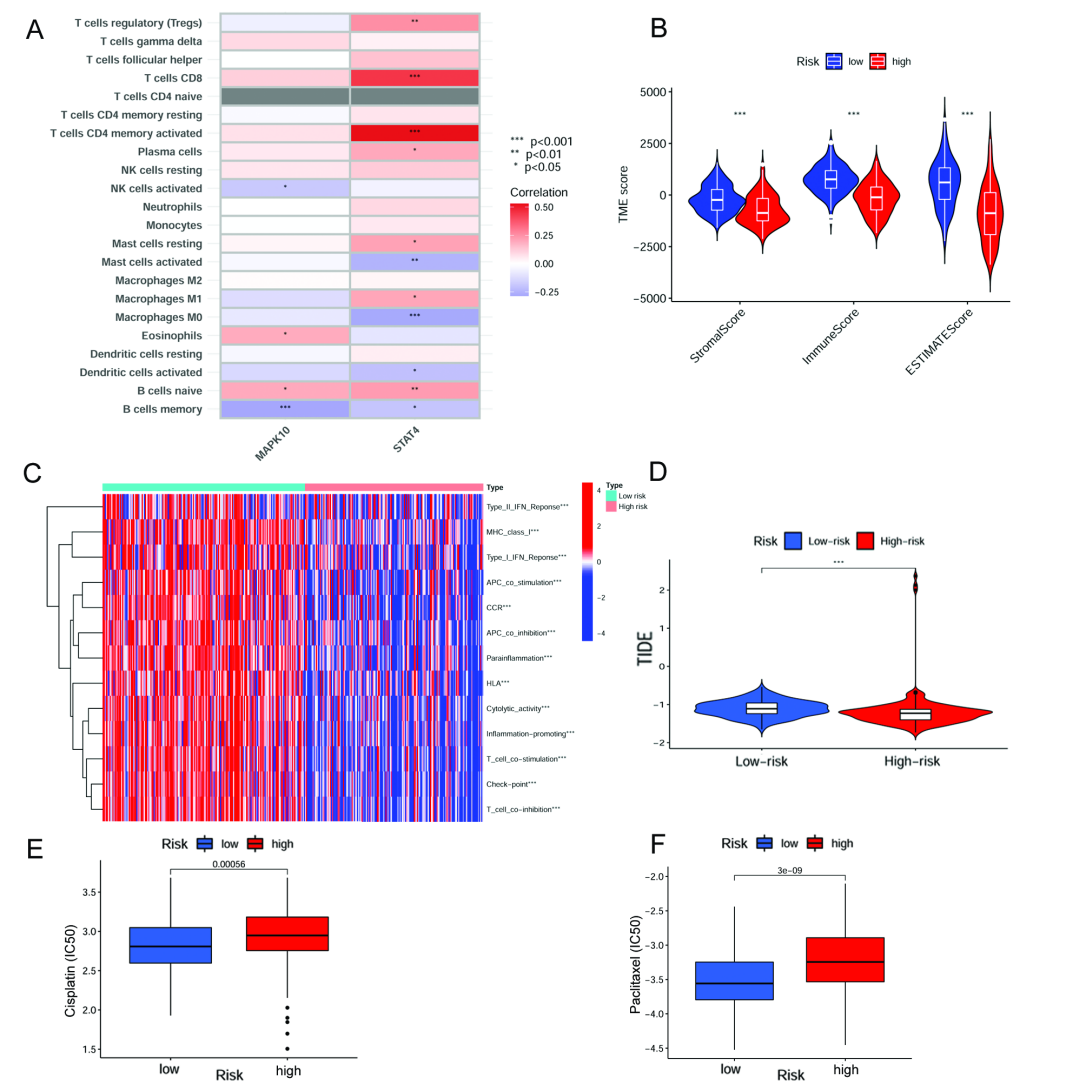
Immune landscape based on the NRG signature. **(A)** Correlations between the immune cell infiltration in OC and two prognostic NRGs in the proposed model. **(B)** TME score in high- and low-risk groups. **(C)** Immune functions enriched in high- and low-risk groups. **(D)** TIDE score between high- and low-risk groups. **(E)** IC50 differences in cisplatin and **(F)** paclitaxel

According to the results of biological pathways, we further explore the enriched immune function and the immune infiltration status in high- and low-risk groups. Firstly, we investigated the correlation between immune infiltration cells and the two-gene signature using the CIBERSORT algorithm. The results showed that the expression of MAPK10 was positively correlated with Eosinophils and B cells naïve infiltration. In contrast, it was negatively correlated with NK cells activated and B cells memory infiltration. The expression of STAT4 was positively correlated with T cells regulatory (Tregs), T cells CD8, T cells CD4 memory activated, Plasma cells, Mast cells resting, Macrophages M1 and B cells naïve infiltration, whereas negatively correlated with Mast cells activated, Macrophages M0, Dendritic cells activated and B cells memory infiltration (Fig. 7A). The risk score was positively correlated with Macrophages M0 and Mast cells activated infiltration. In contrast, it was negatively correlated with Macrophages M1, T cells CD4 memory activated, T cells CD8, and T cells regulatory (Tregs) infiltration (Supplementary Fig. 3). Regarding TME score evaluation, stromal, immune, and ESTIMATE scores were higher in the low-risk group (Fig. 7B). In summary, the correlation between the necroptosis-related risk scores and tumor-infiltrating immune cells was assessed. The results suggested that the risk score was related to different proportions of immune infiltration cells in OC. Subsequently, our team investigated the enriched immune-related functions in the high- and low-risk groups. The results indicated that Type-II IFN Response, MHC class-I, Type-I IFN Response, APC co-stimulation, CCR, APC co-inhibition, Parainflammation, HLA, Cytolytic activity, Inflammation − promoting, T-cell co-stimulation, Checkpoint and T-cell co − inhibition were dramatically different in the two groups (Fig. 7C). Additionally, by comparing to the high-risk group, the TIDE scores were significantly higher in the low-risk group (Fig. 7D).

### Correlation analysis of TFs and prognostic NRGs

To understand the regulating mechanisms of the two prognostic genes, we further examined the expression profiles of TFs in normal ovarian tissue and OC. As a result, we found TFs differentially expressed, including 34 up-regulated TFs and 41 down-regulated TFs. We then investigated the expression levels of the two genes and TF expression, and the potential TFs regulating MAPK10 and STAT4 were shown in (Supplementary Table 4).

### Exploration of therapeutic drug sensitivity

By exploring drug sensitivity, we found that cisplatin and paclitaxel, which present the first-class chemotherapy regimens in OC, were more sensitive in the low-risk group (Fig. 7E **and F**). Moreover, correlations between the risk score and drug sensitivity were displayed in Supplementary Fig. 4, and IC50 in these drugs was also investigated (Supplementary Fig. 5).

## Discussion

Cell death is vitally essential to maintain homeostasis in the cell biological process, which contributes to protecting cells from excessive proliferation. The newly discovered programmed-cell death, including ferroptosis, pyroptosis, and necroptosis, is important in cancer progression [33]. Necroptosis is a caspases-independent cell death mode [34]. This type of death has attracted mounting studies to explore the underlying mechanisms and pathways in multiple cancers. Recent studies have constructed a necroptosis-related risk model based on cancer sequencing analysis. Zhu et al. built an eight necroptosis-related lncRNAs signature with significant values in predicting prognostic in OC [35]. Besides, Yi-Bo He et al. applied six necroptosis-associated lncRNAs to construct a model, which was used to differentiate between hot and cold tumors and provide the treatment strategy for OC [36]. These studies suggested that necroptosis-related lncRNA signature could perform well in predicting prognosis in OC patients. However, genome sequencing is not a popular method in clinical applications, and there may be some difficulties in getting necroptosis-related lncRNA expression in surgical samples of OC patients. If it is possible to develop a risk model based on gene expression, with could be quantified by gene sequencing or immunohistochemical testing, it would be more beneficial for the risk assessment of patients. In our present study, we systematically explored the NRGs expression in OC. More than one-fifth of necroptosis genes (42/204) were differentially expressed between the OC and normal ovarian tissues. Then, we built a two-gene risk model to predict the prognosis for OC patients. MAPK10 and STAT4 were identified as prognostic genes contributing to this model.

NRGs in this risk signature have been widely studied in cancers. MAP kinases act as integration points for various biochemical processes and participate in multiple cellular signals, including cell proliferation, differentiation, and transcription regulation [37]. Knocking down MAPK10 suppressed OC cell growth and migration [38]. The Signal transducer and activator of transcription (STAT) is a prominent transcription factor. STAT family plays a critical pro-tumorigenic role in cancers. A previous study demonstrated that STAT4 is critically involved in gastric cancer metastasis [39]. Cheng et al. indicated that increased expression of STAT4 is tightly associated with cancer cell growth and invasion in colorectal cancer [40]. Zhao et al. reported that STAT4 promotes ovarian cancer metastasis via tumor-derived Wnt7a-induced activation of cancer-associated fibroblasts [41]. However, the high expression of STAT4 was reported to be significantly related to a favorable survival outcome in OC [42, 43]. The results of our study are consistent with these previous findings. The role of MAPK10 and STAT4 in cancer cell necroptosis has been unidentified. The programmed cell death mediated by MAPK10 and STAT4 in ovarian cancer deserves further study.

The uni-Cox and multi-Cox analyses showed that age and risk score were independent risk factors for OC. ROC and C-index curves of risk score and the other clinical variables were plotted to validate the prognostic accuracy. The ROC curve results suggested that age and risk score could be applied as a criterion to predict the survival rate, and the risk score had higher prediction accuracy in 5-year OS. As well as the results of the C-index curve revealed that age could be a more significant risk factor in five years, whereas the prognosis accuracy was more dependent on the risk score after five years. As the results showed by C-index and ROC curves, age is a confounding factor in this model. To reduce the impact of the confounding factor on prognosis prediction, we built a nomogram to predict the survival outcomes of OC patients. This nomogram could comprehensively score patients and assess their survival status at different times. The calibration curves showed excellent agreement between predictions and actual results. Moreover, survival analyses were performed in different clinical subgroups. The risk model behaved well in predicting survival prognosis for patients older and younger than 60 years old, as well as a tumor in grade 3 or stage III-IV. A possible interpretation of the OC patients with grade 1–2 or stage I-II might be the limitations on sample size for OC commonly exhibiting a high degree of malignancy. The results of PCA suggested that the two NRGs had the best capacity to distinguish well between low- and high-risk patients.

The results of GO analysis suggested that NRGs were associated with the interaction between immunoglobulin and antigen, which was a primary process in triggering the immune response. Programmed necrotic cells release their contents and elicit active immune responses from non-immune and immune cells [44]. Nowadays, NRGs associated immune response is rarely studied in cancers. Previous studies have confirmed that cells undergoing necroptosis are involved in immune response activation, particularly antigen presentation and cross-priming of CD8 + T cells [45]. Tania Løve Aaes et al. reported that damage-associated molecular patterns released by necroptotic cancer cells could promote the maturation of dendritic cells, cross-priming of cytotoxic T cells, and the production of IFN-γ in response to tumor antigen stimulation [46]. Meanwhile, KEGG analysis also revealed that NRGs could play a role in Th1 and Th2 cell differentiation, Antigen processing and presentation, Th17 cell differentiation, cytokine − cytokine receptor interaction, and Natural killer cell-mediated cytotoxicity pathways. Th1/Th2 imbalance was involved in necroptosis-mediated inflammation [47]. RIPK1 inhibition could down-regulate Th1 and Th17 cell levels but promote Th2 and Treg cell levels in collagen-induced arthritis [48]. We suspected that necroinflammation could be crucial in TME. The underlying mechanisms mediated by NRGs in OC deserve further research.

Typically, immune infiltration cell in the TME varies with cancer progression. The risk score was positively correlated with Macrophages M0 and Mast cells activated infiltration and negatively correlated with Macrophages M1, T cells CD4 memory activated, T cells CD8, and Treg cells infiltration. A previous study demonstrated that the proportions of mast cells were more remarkable in OC than in benign ovarian neoplasms [49]. More evidence is needed to validate the prognosis value of mast cells infiltrating in OC. Antonio Macciò et al. found that OC patients with a higher M1/M2 ratio present a better survival prognosis than other patients [50]. Treg cell infiltration could suppress protective anti-tumor immune responses, and their accumulation into the TME correlates with a lower survival rate in OC patients [51]. CD4 + and CD8 + T cells were reported to recognize apoptotic OC antigens and exert an anti-tumor effect [52]. The characteristics of immune infiltrating cells correlation calculated by necroptosis-related signature were consistent with the study mentioned above and demonstrated the reliability of the gene signature. We also compared the immune, stromal, and ESTIMATE scores in the two groups and found higher immune scores and lower tumor purity in the low-risk group. Immunotherapy has been applied successfully in malignant tumors, whereas not all OC patients can benefit from immunotherapy. Therefore, it is essential to investigate appropriate biomarkers to select patients with sensitive responses to this treatment. Previous studies have reported that the TIDE algorithm was applied to evaluate the clinical response of patients to ICI treatment. A higher TIDE score can present a greater likelihood of immune escape, indicating a limited response and a worse survival rate for patients treated with ICI. Compared to the low-risk group, patients in the high-risk group had lower TIDE scores, indicating a better immune checkpoint blockade response in OC.

As TMB develops as a potential biomarker for identifying patients likely to respond to ICI, more relevant research has been investigated to characterize the type and the extent of TMB variation across tumor types and histologies [53]. In this research, the risk score was observed to have a negative correlation with TMB, suggesting that low-risk patients can benefit more from immunotherapy. The overall level of TMB can represent the level of effective immune activation, which produces effective new antigens caused by the difference in mutant genes. However, if this change is caused by cell necroptosis remains to be determined. The somatic mutation of NRGs showed that the OC patients in the low-risk group possessed a higher mutation frequency in TP53. TP53 mutations were identified in nearly all serous ovarian tumors [54]. Analysis of standard taxane- and platinum-based chemotherapy-treated OC in the TCGA cohort concerning TP53 mutation types revealed higher rates of chemoresistance in patients with oncomorphic TP53 mutations [55]. Besides, higher CSMD3 mutation frequency was found in the low-risk group. However, previous analyses performed with patients from the TCGA revealed that those with CSMD3 mutation had an OS inferior to those with wild-type CSMD3 [56]. This conclusion was inconsistent with our findings. We speculated it was associated with a different type of mutation, which causes the difference in effective new antigens. It suggested that a single index in the complex regulatory network of the tumor may not be intuitive for prognosis prediction. Thus, the risk score can be used as a more efficient index for predicting prognosis. We investigated the drug sensitivity and found that cisplatin and paclitaxel showed different IC50 between high- and low-risk groups. OC patients in the low-risk group were more sensitive to chemotherapy. Moreover, other potentially sensitive drugs for OC patients in the low-risk group were also revealed in our study.

There are some limitations in the present study. First, it is not the first research that constructed a risk model based on NRGs. Wang et al. reported a risk model consisting of five NRGs [57]. The overall design of Wang’s study was similar to the present study, except for a slightly different approach to constructing the risk model. Wang et al. built a well-performing five-gene model based on necroptosis-related genes and also revealed a close correlation between TME and immunotherapy. Compared with this study, the present study obtained more NRGs (204 genes vs. 75 genes) for comprehensive analysis and risk model construction. In addition, Although Wang’s study built a five-gene risk model behaving well in prognostic prediction, the role of some genes, such as UBD, ATP1A3, and HLA-DOB, remains unclear in ovarian cancer. These genes are worth further investigation. In contrast, there are more defined roles of MAKP10 and STAT4 in ovarian cancer. Meanwhile, MAPK10 and STAT4 have been widely used in pathology work. It is feasible to quantify the gene expression levels by Immunohistochemistry and polymerase chain reaction, which suggests clinical translation potential. Secondly, lacking verification in external datasets is a deficiency of our study. We will contribute to collecting more sample data in our center to certify the prognosis ability of the risk model.

## Conclusion

A well-validated necroptosis-related risk model was built based on two genes, including MAPK10 and STAT4. According to this risk model, the OC patients in the high-risk group were related to worse survival outcomes. The lower TME score was demonstrated in the high-risk group. Patients with lower TMB in the low-risk group showed the best prognosis, and a lower TIDE score suggested a better ICI response in the high-risk group. Besides, we found cisplatin and paclitaxel were more sensitive in the low-risk group. These findings provided novel ways of OC prognosis estimation and potential treatment strategy.

## Electronic supplementary material

Below is the link to the electronic supplementary material.

**Supplementary Material 1:** 204 NRGs were introduced to the present study

**Supplementary Material 2:** The DE-NRGs identified between OC and normal ovarian tissues

**Supplementary Material 3:** The correlation between the clinical variable and the risk score

**Supplementary Material 4:** The correlation between expression of the two prognostic genes and TFs

**Supplementary Material 5:** Identification of prognostic NEGs in OC. **(A)** The seven prognostic NRGs screened out by the uni-Cox regression analysis. **(B)** The 10-fold cross-validation for tuning parameter extraction by the LASSO regression analysis. **(C)** The LASSO coefficient profile of seven prognosis-related NRGs

**Supplementary Material 6:** Functional annotations between high- and low-risk groups. **(A)** The GO analysis of DEGs between the two groups. **(B)** The KEGG pathway analysis of DEGs between the two groups

**Supplementary Material 7:** Correlations of risk score and immune cell types in OC

**Supplementary Material 8:** Correlations of risk score and the sensitive drug in OC

**Supplementary Material 9:** IC50 difference in the potentially sensitive drug in OC

## Data Availability

Publicly available datasets were analyzed in this study. This data can be found in TCGA-OV and GTEx-OV.

## List of abbreviations

C-index: concordance index(C‐index)
DEGs: differentially expressed genes
DE-NRGs: differentially expressed necroptosis-related genes
DE-TFs: differentially expressed transcription factors
DR: death receptors
FC: fold change
FDR: false discovery rate
GTEx: Genotype-Tissue Expression
GO: Gene Ontology
ICI: immune checkpoint inhibitor
IC50: The half-maximal inhibitory concentration
KEGG: Kyoto Encyclopedia of Genes and Genomes
K-M: Kaplan Meier (K-M)
LASSO: least absolute shrinkage and selection operator
Multi-Cox: multivariate Cox
NRG: necroptosis-related genes
OC: ovarian cancer
OS: overall survival
PCA: principal component analysis
ROC: receiver operating characteristic
TCGA: The Cancer Genome Atlas
TFs: transcription factors
TIDE: Tumour Immune Dysfunction and Exclusion
TMB: tumor mutation burden
TME: tumor microenvironment
Tregs: T cells regulatory
Uni-Cox: univariate Cox
