# Supplementary material for: Development of a necroptosis-related gene signature and the immune landscape in ovarian cancer
Source: J Ovarian Res. 2023 Apr 25;16:82. doi: 10.1186/s13048-023-01155-9 (PMC10127035; doi:10.1186/s13048-023-01155-9)
Supplement: Supplementary file 2 — Supplementary Material 2: The DE-NRGs identified between OC and normal ovarian tissues [file 13048_2023_1155_MOESM2_ESM.docx]

**Supplementary Table 2**

| Gene | log2FC | FDR |
| --- | --- | --- |
| TRAF5 | -1.06562 | 1.86E-44 |
| CYLD | -1.26335 | 4.23E-47 |
| CFLAR | -1.13253 | 4.23E-47 |
| CYBB | 1.209258 | 7.32E-29 |
| CAMK2A | -2.9359 | 1.39E-40 |
| CAMK2D | -1.09231 | 4.57E-47 |
| SLC25A31 | -1.60849 | 2.38E-06 |
| GLUD2 | 1.454995 | 8.80E-36 |
| PYGM | -1.87138 | 1.15E-38 |
| MAPK10 | -3.12265 | 4.23E-47 |
| JMJD7-PLA2G4B | -2.9247 | 4.23E-47 |
| PLA2G4B | -4.9507 | 4.23E-47 |
| PLA2G4C | -2.04772 | 3.14E-45 |
| MLKL | -1.03441 | 1.62E-33 |
| IL1B | 1.692205 | 6.37E-29 |
| CHMP4A | -2.02656 | 4.23E-47 |
| CHMP4C | 2.890021 | 3.10E-45 |
| TRPM7 | -1.10121 | 4.23E-47 |
| IL1A | 3.195722 | 1.99E-37 |
| TNFRSF10A | 1.34335 | 1.12E-36 |
| FASLG | 2.941745 | 1.48E-27 |
| IFNB1 | 2.399471 | 1.07E-16 |
| IFNG | 2.20595 | 1.97E-15 |
| JAK2 | -1.31621 | 6.84E-47 |
| JAK3 | -1.10295 | 5.38E-43 |
| STAT4 | -1.41867 | 1.60E-33 |
| STAT5B | -1.18246 | 4.23E-47 |
| IRF9 | -1.68508 | 4.23E-47 |
| TICAM2 | -4.64088 | 4.23E-47 |
| ZBP1 | 1.634347 | 1.14E-17 |
| BCL2 | -1.40335 | 5.71E-44 |
| TSC1 | -1.01074 | 4.55E-47 |
| SIRT3 | -1.05592 | 4.23E-47 |
| DIABLO | -1.64636 | 4.23E-47 |
| CDKN2A | 2.925181 | 3.14E-45 |
| PLK1 | 1.262028 | 3.49E-45 |
| BACH2 | -1.57744 | 5.15E-33 |
| GATA3 | 2.061944 | 2.35E-30 |
| MYCN | 1.37057 | 7.47E-25 |
| ALK | 2.356541 | 2.78E-20 |
| TERT | 5.410726 | 2.09E-43 |
| KLF9 | -1.14748 | 2.30E-44 |
