# Supplementary material for: Development of a necroptosis-related gene signature and the immune landscape in ovarian cancer
Source: J Ovarian Res. 2023 Apr 25;16:82. doi: 10.1186/s13048-023-01155-9 (PMC10127035; doi:10.1186/s13048-023-01155-9)
Supplement: Supplementary file 3 — Supplementary Material 3: The correlation between the clinical variable and the risk score [file 13048_2023_1155_MOESM3_ESM.docx]

**Supplementary Table 3**

| Covariates | Type | Total | Test | Train | p-value |
| --- | --- | --- | --- | --- | --- |
| Age | <=60 | 193(55.14%) | 94(53.71%) | 99(56.57%) | 0.6673 |
|  | >60 | 157(44.86%) | 81(46.29%) | 76(43.43%) |  |
| Grade | G1 | 1(0.29%) | 0(0%) | 1(0.57%) | 0.6451 |
|  | G2 | 41(11.71%) | 23(13.14%) | 18(10.29%) |  |
|  | G3 | 298(85.14%) | 147(84%) | 151(86.29%) |  |
|  | unknown | 10(2.86%) | 5(2.86%) | 5(2.86%) |  |
| stage | Stage I-II | 21(6%) | 12(6.86%) | 9(5.14%) | 0.2965 |
|  | Stage III-IV | 327(93.43%) | 163(93.14%) | 164(93.71%) |  |
|  | unknown | 2(0.57%) | 0(0%) | 2(1.14%) |  |
