# Supplementary material for: Development of a necroptosis-related gene signature and the immune landscape in ovarian cancer
Source: J Ovarian Res. 2023 Apr 25;16:82. doi: 10.1186/s13048-023-01155-9 (PMC10127035; doi:10.1186/s13048-023-01155-9)
Supplement: Supplementary file 4 — Supplementary Material 4: The correlation between expression of the two prognostic genes and TFs [file 13048_2023_1155_MOESM4_ESM.docx]

**Supplementary Table 4**

| Transcription factors | Necroptosis-related gene | Correlation | p-value |
| --- | --- | --- | --- |
| BACH2 | MAPK10 | 0.368625 | 1.76E-13 |
| RFX2 | MAPK10 | 0.2899 | 1.12E-08 |
| HOXB13 | MAPK10 | 0.278438 | 4.37E-08 |
| EBF1 | MAPK10 | 0.26957 | 1.20E-07 |
| TAL1 | MAPK10 | 0.268059 | 1.41E-07 |
| EHF | MAPK10 | -0.25816 | 4.15E-07 |
| NR2F1 | MAPK10 | 0.25432 | 6.23E-07 |
| STAT5B | MAPK10 | 0.247301 | 1.29E-06 |
| NR5A2 | MAPK10 | 0.24558 | 1.53E-06 |
| TET2 | MAPK10 | 0.240787 | 2.47E-06 |
| GATA2 | MAPK10 | 0.23632 | 3.83E-06 |
| E2F7 | MAPK10 | 0.228519 | 8.05E-06 |
| SOX17 | MAPK10 | -0.22246 | 1.41E-05 |
| RBP2 | MAPK10 | 0.221463 | 1.54E-05 |
| TP73 | MAPK10 | 0.2072 | 5.40E-05 |
| PPARG | MAPK10 | 0.199575 | 0.000102 |
| SCML2 | MAPK10 | 0.196965 | 0.000126 |
| HDAC6 | MAPK10 | 0.185905 | 0.000301 |
| HNF4G | MAPK10 | 0.183199 | 0.000369 |
| TEAD4 | MAPK10 | -0.18263 | 0.000385 |
| CHD1 | MAPK10 | 0.182034 | 0.000403 |
| MYH11 | MAPK10 | 0.178041 | 0.000542 |
| EP400 | MAPK10 | 0.172563 | 0.000805 |
| ERG | MAPK10 | 0.17212 | 0.00083 |
| ASCL1 | MAPK10 | 0.169053 | 0.001031 |
| FOXP2 | MAPK10 | 0.163127 | 0.001549 |
| FOXA1 | MAPK10 | 0.159323 | 0.001997 |
| FOXA2 | MAPK10 | 0.157325 | 0.002278 |
| KLF4 | MAPK10 | 0.156073 | 0.002472 |
| MITF | MAPK10 | 0.152543 | 0.003101 |
| TCF21 | MAPK10 | 0.150891 | 0.003443 |
| NR4A1 | MAPK10 | 0.14976 | 0.003697 |
| FLI1 | MAPK10 | 0.144476 | 0.00512 |
| FOXP1 | MAPK10 | 0.138047 | 0.007505 |
| PBX3 | MAPK10 | 0.13048 | 0.011546 |
| TFAP2C | MAPK10 | -0.12995 | 0.011893 |
| KLF5 | MAPK10 | -0.12283 | 0.017483 |
| ELL2 | MAPK10 | 0.114453 | 0.026878 |
| GATA4 | MAPK10 | 0.109051 | 0.035016 |
| SALL4 | MAPK10 | 0.105132 | 0.042156 |
| BATF | MAPK10 | -0.10276 | 0.047041 |
| NCAPG | MAPK10 | 0.101609 | 0.049584 |
| STAT4 | STAT4 | 1 | 0 |
| EOMES | STAT4 | 0.641971 | 7.83E-45 |
| ELL2 | STAT4 | 0.552811 | 2.57E-31 |
| FOXP3 | STAT4 | 0.552625 | 2.72E-31 |
| FLI1 | STAT4 | 0.509757 | 3.97E-26 |
| BATF | STAT4 | 0.424587 | 8.43E-18 |
| CEBPA | STAT4 | 0.395896 | 1.75E-15 |
| SPIB | STAT4 | 0.394141 | 2.38E-15 |
| MEF2C | STAT4 | 0.381994 | 1.93E-14 |
| MAF | STAT4 | 0.373182 | 8.38E-14 |
| SOX17 | STAT4 | -0.37159 | 1.09E-13 |
| PBX3 | STAT4 | 0.276296 | 5.59E-08 |
| ERG | STAT4 | 0.267778 | 1.46E-07 |
| MITF | STAT4 | 0.265088 | 1.96E-07 |
| NR5A2 | STAT4 | 0.246287 | 1.42E-06 |
| GATA3 | STAT4 | 0.231083 | 6.32E-06 |
| VDR | STAT4 | 0.227921 | 8.52E-06 |
| PPARG | STAT4 | 0.22677 | 9.48E-06 |
| FOXA1 | STAT4 | 0.219236 | 1.89E-05 |
| CBX7 | STAT4 | 0.20628 | 5.84E-05 |
| ARNTL | STAT4 | 0.185601 | 0.000308 |
| KLF4 | STAT4 | 0.179466 | 0.000488 |
| FOXP1 | STAT4 | 0.1762 | 0.000619 |
| GREB1 | STAT4 | -0.17024 | 0.000948 |
| MAFF | STAT4 | 0.167026 | 0.001186 |
| C17orf96 | STAT4 | -0.16594 | 0.001278 |
| TAL1 | STAT4 | 0.164262 | 0.001434 |
| NFE2 | STAT4 | -0.15837 | 0.002126 |
| EBF1 | STAT4 | 0.148519 | 0.003995 |
| TET2 | STAT4 | 0.1426 | 0.005733 |
| STAT5B | STAT4 | 0.132986 | 0.010034 |
| FOXP2 | STAT4 | 0.122137 | 0.018129 |
| RFX2 | STAT4 | -0.11086 | 0.032085 |
| SPDEF | STAT4 | -0.11071 | 0.032315 |
| FOXA2 | STAT4 | -0.10976 | 0.033838 |
| RUNX1T1 | STAT4 | 0.104535 | 0.043343 |
| KDM4C | STAT4 | 0.103967 | 0.0445 |
| MYH11 | STAT4 | 0.1029 | 0.046744 |
| TFAP2C | STAT4 | 0.101668 | 0.049451 |
