# Supplementary figures and images for: Development of a necroptosis-related gene signature and the immune landscape in ovarian cancer
Source: J Ovarian Res. 2023 Apr 25;16:82. doi: 10.1186/s13048-023-01155-9 (PMC10127035; doi:10.1186/s13048-023-01155-9)

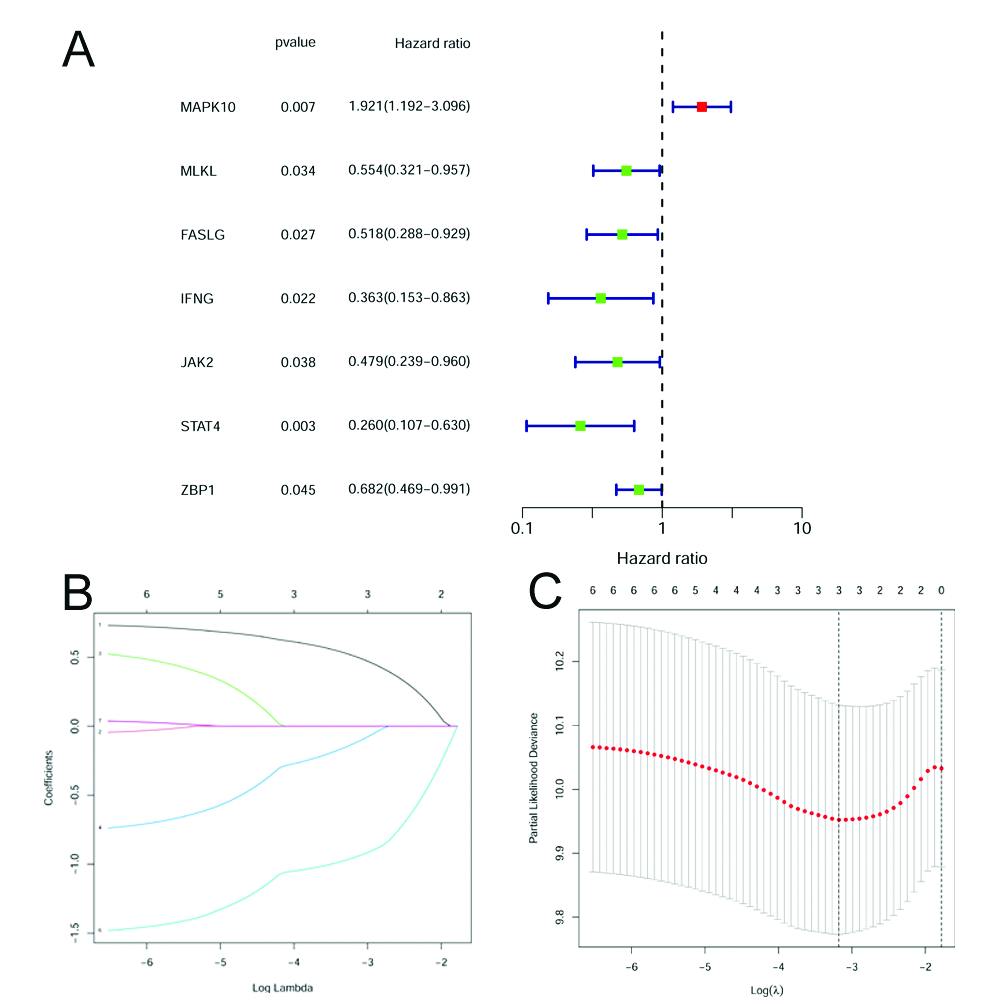

Supplement: Supplementary file 5 — Supplementary Material 5: Identification of prognostic NEGs in OC. (A) The seven prognostic NRGs screened out by the uni-Cox regression analysis. (B) The 10-fold cross-validation for tuning parameter extraction by the LASSO regression analysis. (C) The LASSO coefficient profile of seven prognosis-related NRGs [file 13048_2023_1155_MOESM5_ESM.png]

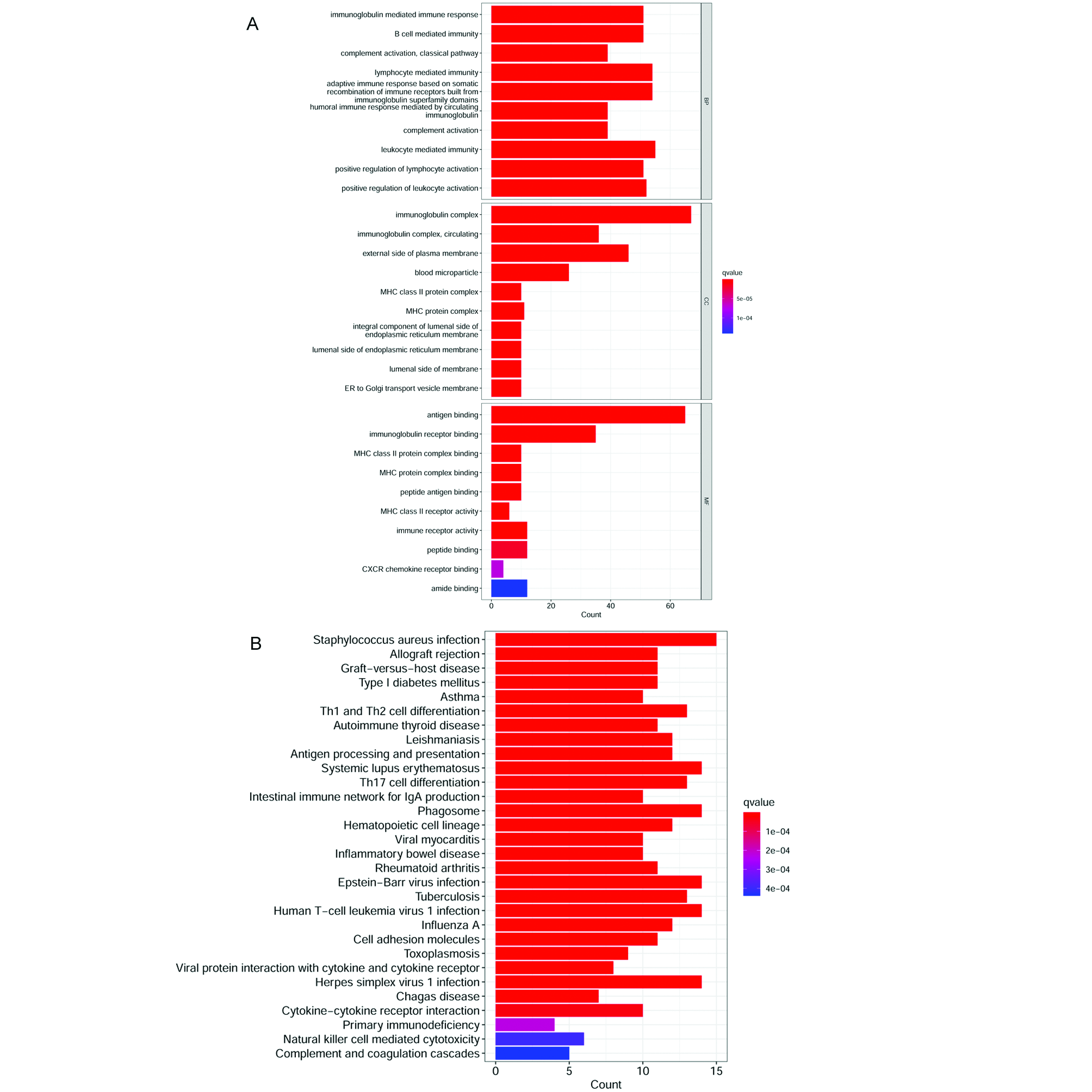

Supplement: Supplementary file 6 — Supplementary Material 6: Functional annotations between high- and low-risk groups. (A) The GO analysis of DEGs between the two groups. (B) The KEGG pathway analysis of DEGs between the two groups [file 13048_2023_1155_MOESM6_ESM.png]

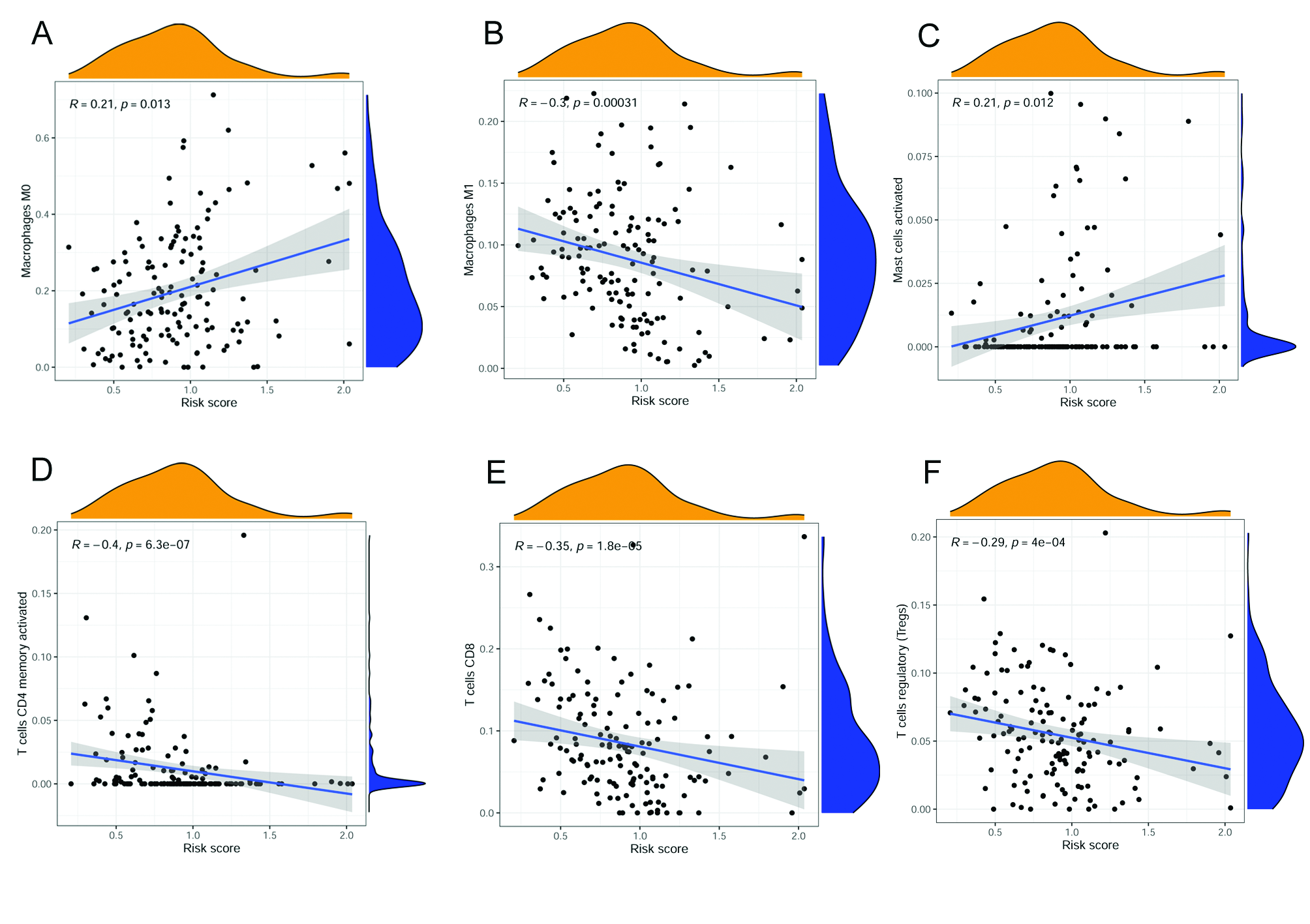

Supplement: Supplementary file 7 — Supplementary Material 7: Correlations of risk score and immune cell types in OC [file 13048_2023_1155_MOESM7_ESM.png]

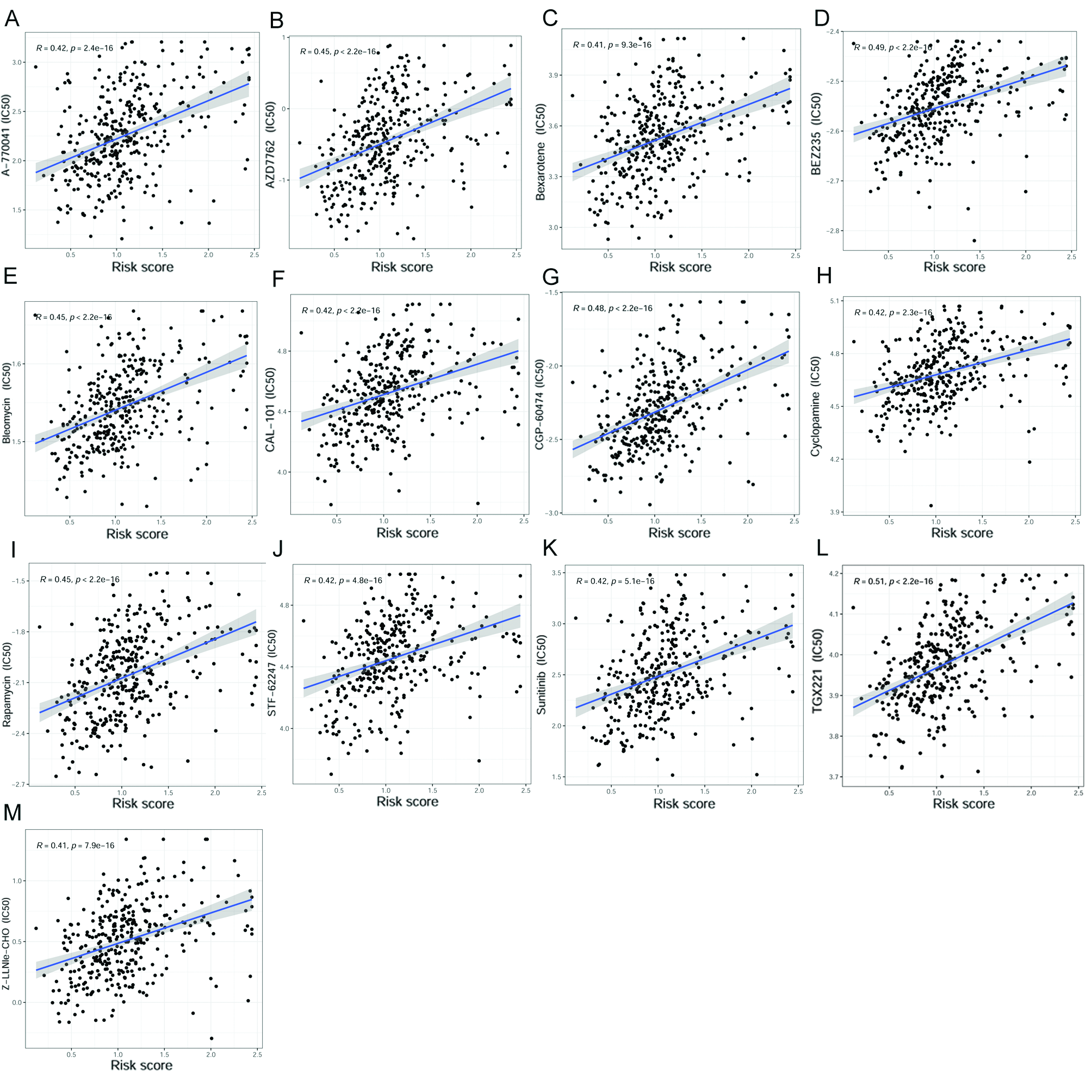

Supplement: Supplementary file 8 — Supplementary Material 8: Correlations of risk score and the sensitive drug in OC [file 13048_2023_1155_MOESM8_ESM.png]

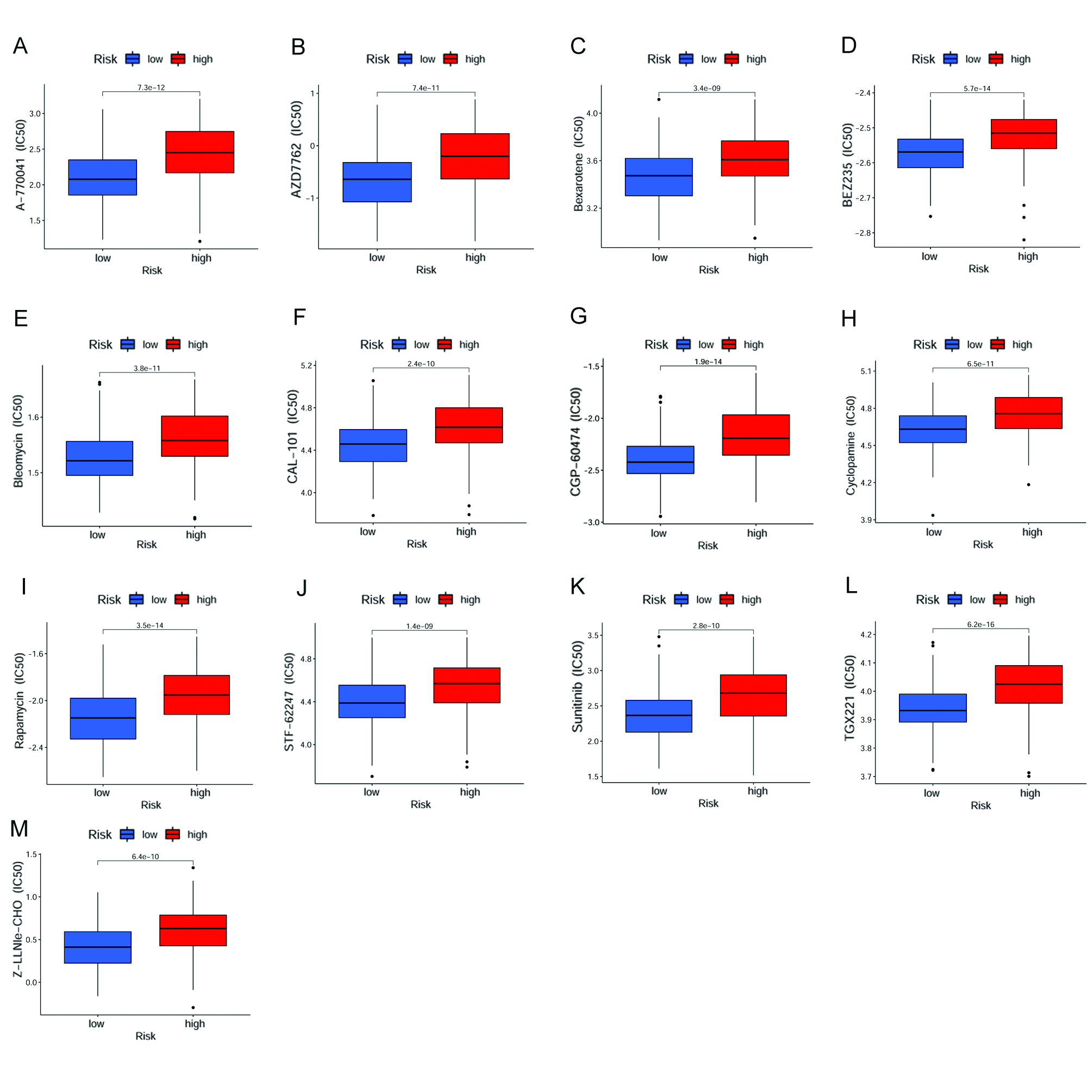

Supplement: Supplementary file 9 — Supplementary Material 9: IC50 difference in the potentially sensitive drug in OC [file 13048_2023_1155_MOESM9_ESM.png]
